# An Innovative Stereolithography 3D Tubular Method for Ultrathin Polymeric Stent Manufacture: The Effect of Process Parameters

**DOI:** 10.3390/polym15214298

**Published:** 2023-11-01

**Authors:** Aniol Bosch, Enric Casanova-Batlle, Iuliana Constantin, Carles Rubio, Joaquim Ciurana, Antonio J. Guerra

**Affiliations:** 1 Eurecat, Technology Centre of Catalonia, 08290 Cerdanyola del Vallès, Spainiuliana.constantin@eurecat.org (I.C.); carles.rubio@eurecat.org (C.R.); 2 Departament of Mechanical Engineering and Industrial Construction, University of Girona, Maria Aurèlia Capmany 61, 17003 Girona, Spain; enric.casanova@udg.edu

**Keywords:** additive manufacturing, 3D printing, stereolithography, bioresorbable stent, medical devices

## Abstract

In the last decades, researchers have been developing bioresorbable stents (BRS) to overcome the long-term complications of drug-eluting stents (DES). However, BRS technology still presents challenging limitations in terms of manufacturing, materials, or mechanical properties. At this juncture, companies have developed ultrathin DES that may further improve the efficacy and safety profile of traditional DES by reducing the risk of target-lesion and target-vessel failures until BRS are developed. Nonetheless, the metallic platform of ultrathin DES still presents problems related to their cellular response. The use of polymers as a permanent platform in DES has not previously been studied due to the limitations of current manufacturing technologies. In this work, an innovative manufacturing method for polymeric stent production using tubular stereolithography (SLA) technology is proposed both for BRS and for ultrathin polymeric DES. The effects of manufacturing process parameters were studied by modelling the outcomes (stent thickness and strut width) with the key manufacturing variables (exposure, resin volume, and number of layers). Two different laser setups were used to compare the results. Microscopy results proved the merit of this novel tubular SLA process, which was able to obtain stents with 70 μm strut width and thickness in barely 4 min using only 0.2 mL of resin. Differential Scanning Calorimetry (DSC) results showed the stability of the manufacturing method. The results obtained with this innovative technology are promising and overcome the limitations of other previously used and available technologies.

## 1. Introduction

One of the leading causes of morbidity and mortality worldwide is coronary artery disease, a condition characterized by the narrowing of the artery due to plaque deposits [1]. Stents are small medical devices that allow the restoration of normal blood flow in blood vessels that have become obstructed. Since their introduction into the medical sector, stents have saved millions of lives. Stents have evolved since their introduction. Currently, drug-eluting stents (DES) are the outstanding devices used to treat this pathology. However, the metallic core of DES remains inside the body for the patient’s lifetime and can lead to long-term complications, such as in-stent restenosis and late thrombosis. Because of this, bioresorbable stents (BRS) have gained popularity within the scientific community in the last decade. BRS appear to solve the major problems of DES. Nonetheless, BRS technology still presents challenging limitations in terms of materials, mechanical properties, or their manufacturing process. The scientific community have been analyzing this field over the last decade.

In 2014, Stepak et al. fabricated a 300 μm strut-width stent from a combination of PLLA/PLGA by laser cutting [2], but the stent’s structural characteristics and mechanical properties were significantly compromised by the generation of heat-affected zones due to the laser cutting process. This phenomenon was also studied by Pei-Jiang Wang et al. [3], who directly related macroscopic properties, such as mechanical strength and material degradation, to microscopic structures highly affected by laser-derived heat. Although other types of laser such as femtosecond lasers reduce the thermal effect in polymers, other damages to the surface are inevitable [4,5], making traditional manufacturing processes suboptimal methods for the production of polymer stents.

Like other medical devices, BRS can be manufactured via additive manufacturing (AM) methods. AM provides greater manufacturing speed, the possibility of more complex geometries, and the customization of medical devices to patient-specific needs, as well as reduced manufacturing, supply chain, and inventory costs [6]. In 2011, Park et al. prepared a helical, biocompatible, and biodegradable scaffold by combining a 3D rapid prototyping plotting system with an electrospinning apparatus [7]. A few years later, in 2015, Park et al. fabricated a PCL-based stent with a sirolimus coating and tested it in vivo in castrated pigs [8]. Although the stent was successfully implanted into the porcine femoral artery, it was not dimensionally or mechanically analyzed. Additionally, the technology used did not allow for high personalization of the stents, and it was highly time-consuming [9]. In 2017, Ware et al. [10] used micro-Continuous Liquid Interface Production (μCLIP) to print BRS from bioresorbable photopolymerizable materials, meeting the medical devices’ dimensional requirements. Van Lith et al. [11] also fabricated BRS using the μCLIP system with some modifications and achieved stents with a strut size of 150 μm. Although good results were obtained, and the μCLIP technology allows for continuous production and the avoidance of interlayer times—thus decreasing the time taken by a factor of 100 compared to Direct Light Processing (DLP) [12]—it still took 70 min to fabricate a 20 mm stent. In 2017, Sol et al. fabricated a polymeric self-expandable bioresorbable stent in a Cartesian Fused Deposition Modelling (FDM) 3D printer and evaluated its mechanical and biological properties; the results showed the feasibility of producing polymeric stents comparable to nitinol stents and concluded that additive manufacturing technologies are promising in this context. Nevertheless, the Cartesian approach had limitations in terms of the geometry, dimensional accuracy, and surface quality.

Traditional Cartesian AM technologies have proved their effectiveness; however, Cartesian systems demand lamination of the stent and structural supports to produce the object. This increases the printing time and restricts the mechanical properties of stents. In response, in 2017, Guerra et al. [13] presented for the first time a tubular FDM machine, demonstrating the possibility of cylindrical 3D printing for stent manufacturing. The authors studied the effect of the temperature, printing speed, and polymer flow rate. Zhao et al., in 2019, also proposed a rotating system for an FDM 3D printer to avoid supports during the fabrication, and with which stents with relative shape fidelity could be successfully printed [14]. The work studied three different geometries and their process parameters. In 2022, Chausse et al. [15] developed a versatile solvent-cast direct-write printing system to fabricate BRS on a rotating cylinder using polymer inks. A similar approach was followed by Casanova-Batlle et al. to develop a tubular Direct-Ink Writing (DIW) machine and characterize the process parameters [16]. Despite the results obtained and the possibility of using biocompatible materials, ink technologies have limitations in the fabrication of complex geometries, such as the ones found in stent technology [17]. Tubular FDM technologies have given acceptable results but have not reached the precision required by the medical sector. SLA processes seem to be very effective in terms of precision but have limitations in terms of cost, time, and the development of new biocompatible and bioabsorbable materials [18].

As can be seen, BRS technology still presents challenging limitations in terms of manufacturing but also in terms of materials or mechanical properties. To reach a faster solution, companies have developed ultrathin DES that may further improve the efficacy and safety profile of traditional DES by reducing the risk of target-lesion and target-vessel failures. Nonetheless, the metallic platform of ultrathin DES still presents problems related to their cellular response. The use of polymers as a permanent platform in DES has not previously been studied due to the limitations of current manufacturing technologies. To overcome these limitations and harness the potential of technology for manufacturing stents, a novel tubular SLA system is developed herein to simplify the process and bring previously unattainable benefits to the traditional approach in the ability to produce polymeric stents (permanent or bioresorbable).

This work presents this novel system and studies the fundamental characteristics of the process to propose a mathematical model for the dimensional characterization of the key features of stents obtained through a Design of Experiments (DoE), employing the Response Surface Methodology (RSM) to optimize the experimental parameters and model the novel printing process. A three-factor, five-central-point Box–Behnken Design (BBD) was carried out to study the effect of the printing feedrate, resin volume, and number of layers on the strut width and stent thickness. Two different laser setups were used to improve the results. Microscopy results proved the merit of this novel tubular SLA process, which was able to obtain stents with 70 μm strut width and thickness in barely 4 min using only 0.2 mL of resin. Differential Scanning Calorimetry (DSC) results show the stability of the manufacturing method. The results obtained with this innovative technology are promising and overcome the limitations of other previously used and available technologies.

## 2. Materials and Methods

### 2.1. ST3DT—An Innovative 3D Printing Machine

**Figure 1:**
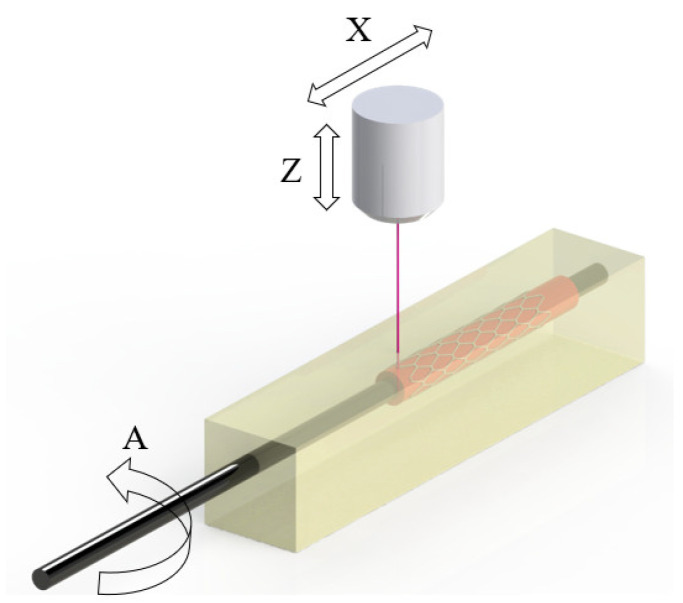
Schematic of the resin vat and build-tube (ST3DT).

An innovative vat polymerization machine to fabricate tubular medical prostheses was developed. The STereolithography 3D Tubular (ST3DT) method is based on vat polymerization technology. This novel system is based on the introduction of a tubular bed, also known as rotary mandrel-assisted printing. This mandrel (A axis) is inside a tank containing photosensitive resin, which is impregnated onto it (Figure 1) by controlling the quantity of resin in the vat and the rotating speed; then, a laser (X, Z axes) scans the desired geometry to obtain a single-layer or multi-layer tubular part. The system allows the fabrication of stents and other tubular prostheses without the need to use supports during the process, unlike all other systems based on vat polymerization. Moreover, this novel approach allows the fabrication of prostheses following trajectories that are impossible with Cartesian systems. Combined with its high precision, this technology offers the possibility of manufacturing stents with highly complex geometries that are impossible to achieve using traditional technologies. In addition, layered lamination, both radially and longitudinally, is eliminated, thus improving the mechanical properties of the prosthesis. Depending on the amount of resin used, the mandrel is totally covered or partially covered, creating an impregnated resin film. With the impregnation system, the amount of resin required is reduced to a minimum, and the possibility of manufacturing with more than one material expands the potential of this technology. Furthermore, as it is an additive manufacturing technology, complex 3D reliefs on the prosthesis surface can be created to provide different properties or patient-specific solutions. Photosensitive resins are one of the most promising materials in the medical field because they can be easily chemically modified, and new formulations can be developed that provide different mechanical and biological properties. The possibility of drug encapsulation in biodegradable resins is a key factor providing an advantage over other technologies to achieve a stent-pill.

The laser installed is a 500 mW Gaussian laser with a wavelength of 405 nm. Two different configurations were used in the experiments to obtain a mathematical model of the direct laser configuration. For the validation of the model and production of the final ultrathin stent, a collimated laser was used.

This novel method was designed, fabricated, and patented (PCT/EP2021/080095) by Eurecat, Technology Centre of Catalonia (Cerdanyola del Vallès, Spain).

A FormLabs Washing Station and FormLabs Cure Station (FormLabs, Somerville, MA, USA) were used to wash and cure the fabricated stent. After printing, the stents were washed for 5 min in IPA and dried prior to curing. The curing process was performed for 30 min at 70 °C. The equipment allowed stents to be cured using 13 multi-directional LEDs, with a total power of 39 W at 405 nm wavelength.

### 2.2. Stent Model

**Figure 2:**
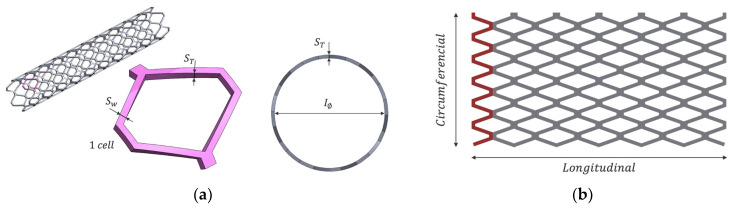
(**a**) CAD stent, detail of a single diamond cell, and the profile of the stent; (**b**) Printing trajectory of the experiment, where the red line shows the first segment printed.

The stent model used for the experiments was a Palmaz-Schatz geometry (Figure 2) cell characterized by the following parameters: 3.4 mm inner diameter ($I_{Ø}$), 20 mm length (L), 6 circumferential cells ($N_{C,\;y}$), 6 longitudinal cells ($N_{C,\;x}$), 100 μm stent thickness ($S_{T}$), and 150 μm strut width ($S_{w}$). A Palmaz-Schatz stent was chosen for its ease of design and fabrication. In addition, it is a highly studied geometry and allows for a thorough comparison with the literature [19]. The Palmaz-Schatz design, based on diamond cells, permits the study of strut width and stent thickness parameters with ease, and due to its high stiffness [20], it can be easily manipulated during analysis. $S_{w}$ refers to the thickness of an individual strut as seen from a top view. On the other hand, $S_{T}$ refers to the thickness in the radial direction, also known as the stent profile, meaning the difference between the outer radius and the inner radius.

The trajectory followed was along the perimeter of the tubular axis and longitudinally forward. The stent model was designed in SolidWorks 2022 (SolidWorks Corp., Waltham, MA, USA).

### 2.3. Materials

A methacrylate-based resin (Surgical Guide (SG), FLSGAM01, FormLabs Ohio, Lemoyne Rd., Suite O Millbury, OH, USA) was used as the base material for stent fabrication. SG is a class I biocompatible resin (Table 1). Isopropyl alcohol (IPA, 96°) (Quimimont, Barcelona, Spain) was used to clean the non-photopolymerized resin present in the fabricated stent prior to curing.

### 2.4. Design of Experiments

Screening experiments were carried out to find the correct process parameter levels to produce stents. A $2^{3}$ full factorial design was used, and the parameters studied were the power (P: 11/80 mW), feedrate (F: 200/800 mm/min), resin volume (V: 0.3/0.6 mL), number of layers (L: 1/2), and temperature (T: 25/45). The method of steepest descent was performed to reach new levels, and a second screening experiment was carried out, leading to new parameter values for the power (P: 11/32/45 mW), feedrate (F: 400/600/800 mm/min), resin volume (V: 0.3/0.45/0.6 mL), number of layers (fixed at 1 layer), and temperature (fixed at 30 °C). Based on the screening experiment results, the Design of Experiments (DoE) printing process parameters were selected based on their effect on the final geometry. By employing a Box–Behnken Design (BBD) DOE with 5 central points and 4 replicas, 64 samples were printed (Table 2).

The samples were analyzed, and $S_{W}$ and $S_{T}$ were measured, as these are critical parameters when fabricating stents. These two parameters directly impact the safety of the stent in both transport and implantation, making them two fundamental research topics. More specifically, thinner strut widths reduce the risk of in-stent restenosis and promote better re-endothelialization [21]. In addition, thinner stents reduce disturbances and improve hemodynamics. All the different stent types, designs, and geometries are defined by different parameters; nevertheless, these two parameters are common to all.

### 2.5. Stereolithography Mathematical Model

From the Beer–Lambert Law, a theoretical relationship between resin and exposure (energy/area) can be developed [22]. As exposure is a function of the power, beam radius, and scan speed of the laser (Equation (1)), the laser beam power was fixed at 10 mW, while modifying the feedrate. The temperature was fixed at 30 °C.

(1) $$E\left(x,y,z\right)=\sqrt{\frac{2}{\pi }}\cdot \frac{P_{L}}{W_{0}V_{s}}\cdot e^{-\frac{2y^{2}}{W_{0}^{2}}}\cdot e^{-\frac{z}{D_{p}}}$$

Here, E is the exposure (energy/unit area), $P_{L}$ is the power of the laser, $W_{0}$ is the radius of the laser beam, $D_{p}$ is the laser penetration depth, and $V_{s}$ is the scan speed of the laser (feedrate). The cure depth ($C_{d}$) determines the depth where energy is sufficient to bring resin to the gel point [23], and it is defined as

(2) $$C_{d}=D_{p}\ln\left[\sqrt{\frac{2}{\pi }}\cdot \frac{P_{L}}{W_{0}V_{s}E_{c}}\right]$$

where $E_{c}$ is the critical exposure (at which resin starts to solidify).

### 2.6. Characterization

#### 2.6.1. Physical and Morphological Analysis

A Nikon SMZ 745T optical microscope (Nikon Instruments Inc., Melville, NY, USA) attached to a CT3 ProgRes digital microscope camera was used to analyze the dimensional parameters of the printed stents. Image J^®^ was used to process the images and collect the data to measure $S_{W}$ and $H_{W}$. A Micrometer Micromar 40 EWV (Mahr, Göettingen, Germany) digital micrometer was used to measure $S_{T}$. To analyze the stent’s surface morphology, the samples were examined via Scanning Electron Microscopy (SEM) using a Hitachi S-4100 (Hitachi Europe Limited Buckinghamshire, Slough, UK). Prior to analysis, the samples were coated with a layer of evaporated carbon in an Emitech K950 turbo evaporator.

#### 2.6.2. Differential Scanning Calorimetry

Thermal analysis of the presented polymers was performed via DSC. The technique was carried out through Q-20 and RCS-90 cooling modules (TA Instruments, Inc., New Castle, DE, USA). Measurements were carried out in the following sequence: Firstly, the sample was cooled to −20 °C and then heated to 200 °C to obtain the degree of curing. Once it reached the maximum curing degree, the sample was cooled to −20 °C and subsequently heated to 200 °C. All the tests were driven with a heating rate of 20 °C/min. During all processes, 50 mL/min of dried and high-purity nitrogen was applied. The samples typically weighed between 2.0 and 7.0 mg and were taken from the inner part of the middle of the specimens fabricated via ST3DT. Each sample was placed in an aluminum crucible. DSC is the most frequently employed thermoanalytical technique for polymer investigation, in which the difference in heat flow rate between a sample and an inert reference is measured as the sample is heated or cooled and determined as a function of time or temperature.

#### 2.6.3. Laser Characterization

The laser beam power was measured using PM16-121 (Thorlabs, Newton, NJ, USA), a standard photodiode power meter ranging from 400 to 1100 nm. The variation of the power measurement was 0.5%, and ambient light was estimated to contribute <0.46 μW. The direct laser used at the working power had a spot radius of 68.5 μm, and the fiber optic and collimator laser had a spot radius of 25 μm.

## 3. Results and Discussion

The experimental results are presented in this section. An analysis of variance (ANOVA) was performed to test the significance of the process parameters and the dimensional outcomes. The ANOVA was applied with 95% confidence (α=0.05), and the parameters fitting this value of significance are marked with an asterisk (*) in the results. The ANOVA was also used to rule out any trend in variance between replicates.

### 3.1. DoE and RSM Model

With regards to $S_{T}$, the ANOVA results revealed that all the main factors and one interaction and quadratic factor significantly influenced $S_{T}$. A quadratic regression model was calculated to fit the experimental data, eliminating the non-influential parameters (p>0.05). The resulting model is shown in Equation (3):

(3) $$S_{T}=0.1042E+0.3963V+0.1585L-0.0624EV-0.0041EL+0.2736VL-0.0053E^{2}+0.1855V^{2}-0.0296L^{2}-0.4799$$

where E is the exposure in [mJ/mm^2^], V is the resin volume in [mL], and L is the number of layers in [ut]. The model showed $R^{2}=0.903$ and $p-\mathrm{value}\;<\;2.2\times 10^{-16}$, indicating a good fit to the experimental data, between the levels studied.

The model pointed out that volume is a key factor for estimating $S_{T}$. The more resin above the tubular axis, with sufficient exposure energy, the higher $C_{d}$ will be, leading to a higher $S_{T}$. For the same reason, as exposure increases, $S_{T}$ is affected in reverse. The results also show the influence of the interaction of the number of layers and the volume, with a proportional increase in $S_{T}$ observed as it increases. This indicates that as more layers are made in the stent, more resin will consolidate in the stent if there is sufficient resin impregnated in the tubular bed.

Concerning $S_{w}$, a quadratic model was calculated to fit the experimental data for strut width, eliminating the non-influential parameters (*p* > 0.05). The interaction effects between exposure and the number of layers showed significance. The resulting model is shown in Equation (4):

(4) $$S_{W}=0.2267E+1.1V-0.068L+0.044EV+0.019EL+0.013VL-0.0145E^{2}-2.0794V^{2}-0.0186L^{2}-0.9825$$

where E is the exposure in [mJ/mm^2^], V is the resin volume in [mL], and L is the number of layers in [ut]. The model showed $R^{2}=0.8417$ and *p*-value $<2.2\times 10^{-16}$, indicating a good fit to the experimental data, between the levels studied.

The model pointed out that exposure and volume are key factors for estimating $S_{W}$. As the feedrate is part of the function for exposure, the higher the speed, the less energy is applied to the same voxel and, therefore, the less resin is cured, leading to a smaller $L_{W}$. The results show that as exposure decreases (printing speed increases), smaller strut widths are achieved. The results also show the influence of the volume, with an increase in the strut width as it increases, within the limits studied.

**Figure 3:**
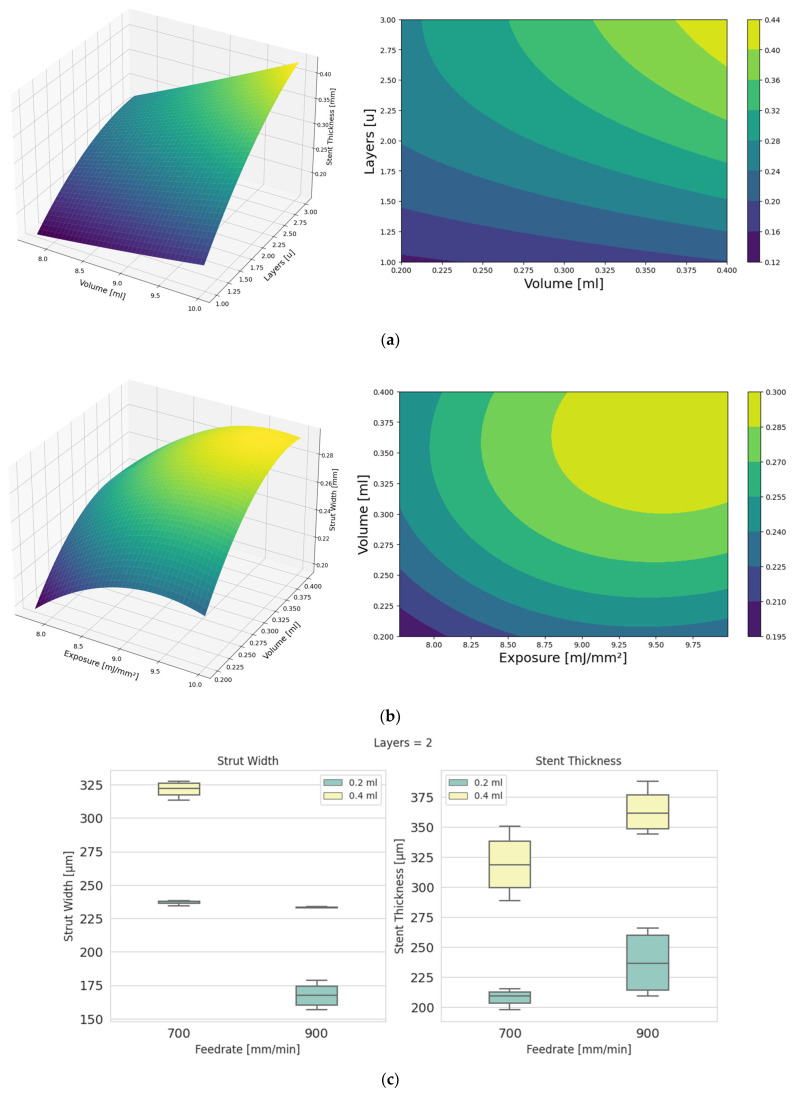
(**a**) The 3D surface response and contour plot for $S_{T}$ (*E* = 8.87 mJ/mm^2^); (**b**) The 3D surface response and contour plot for $S_{W}$ (L = 2); (**c**) Boxplot for $S_{W}$ and $S_{T}$ grouped by *V* and *F*.

The thickness of the stent is highly influenced by the number of layers that are made to fabricate the stent; however, it is also highly influenced by the amount of resin inside the tank, and it is directly related to the thickness of impregnation and the amount of resin that is impregnated—and, therefore, can be cured—or the thickness of resin that submerges the mandrel. The combination of these two parameters allows the stent thickness to be adjusted to the required thickness. The strut thicknesses achieved within the limits studied ranged from 120 μm to 440 μm (Figure 3a).

As long as uncured resin is impregnated into the mandrel, the number of layers in the stent fabrication will be decisive for increasing the thickness when needed; however, the resin volume is decisive in the case of single-layer stents. In the case of strut width, by studying the volume and exposure during printing, the surface response is similar, being highly affected by the exposure received. In this case, widths vary between 195 μm and 300 μm, within the limits studied (Figure 3b). The results for two layers grouped by exposure and by resin volume in the reservoir showed that for the stent thickness, there is a larger variability. In contrast, the strut width showed minimal variability in most cases (Figure 3c).

From Equation (1), the line width of the cured trajectory can be estimated as follows:

(5) $$L_{W}=W_{0}\sqrt{\frac{2C_{d}}{D_{p}}}$$

From Equation (2),

(6) $$L_{W}=W_{0}\sqrt{2\ln\left(\sqrt{\frac{2}{\pi }}\cdot \frac{P_{L}}{W_{0}V_{s}E_{c}}\right)}$$

To compare the Beer–Lambert Law with the RSM model and experimental results, run 6 was used. According to the Beer–Lambert Law, the theoretical strut width should be 199.6 μm, while the RSM model estimated 181.8 μm and the experimental result presented an average of 191.7 μm, giving an error of ±5.3% between the experimental results and mathematical models (Table 3).

Since physical parameters that the Beer–Lambert Law does not contemplate were used to calculate the model, they may be a source of variability. In the case of a single layer, it could be considered as an error, although for stents with more than one layer, according to the experimental results, the model calculation adds precision.

### 3.2. Physical and Morphological Analysis

**Figure 4:**
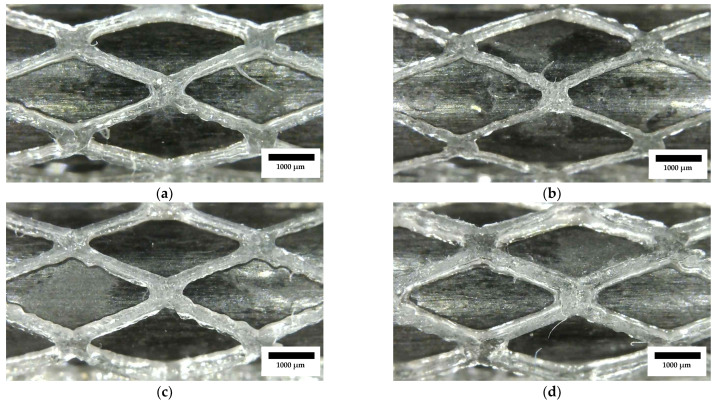
Random samples from the DoE: (**a**) B1-R1; (**b**) B1-R2; (**c**) B1-R8; (**d**) B1-R9. Detail of diamond cells with surface defects and the presence of waves due to the rotational speed of the laser agitating the resin in the tank. Scale bar is 1000 µm.

The optical images (Figure 4) showed dimensional precision and surface properties that do not meet the requirements of stents. Zig-zag patterns were seen in the diagonal sections of the diamond cells, as well as inconsistent strut widths. These effects could have been produced by the direct laser configuration employed in the experiments. This configuration does not allow lower power, and for this reason, the minimum exposure of the resin in the tank was restricted. One strategy available to reduce the exposure was to increase the speed, but it was already demonstrated that rotating the tubular bed faster would remove too much resin and promote the creation of waves on the stent surface and lower geometric precision. In response to all these observations, the laser setup was changed to a fiber optic laser. A fiber optic assembly with a collimator allows a more concentrated light to be obtained so that a smaller light beam radius is achieved; with the loss of power, this gives the ability to use smaller exposures and print at a lower speed than with the direct laser. In addition, the difference in mass between the collimator and the laser is substantial, which reduces the inertia in the machine during printing and benefits the final precision, eliminating all the waves on the surface and the zig-zag patterns in the trajectories (Figure 4).

### 3.3. Collimated Laser

This section aims to validate the RSM model and the production of ultrathin stents. From Equation (1), the maximum exposure ($E_{\max}$) depends on $P_{L}$, $W_{0}$, and $V_{s}$.

(7) $$E_{max}=\sqrt{\frac{2}{\pi }}\cdot \frac{P_{L}}{W_{0}V_{s}}$$

By matching the exposure from the direct laser to the fiber optic laser, the model should describe the system response. With the direct laser, the exposure studied in the DoE was between 7.76 and 9.98 mJ/mm^2^, with $W_{0}$ = 68.5 μm, $V_{s}$ = 700~900 mm/min, and $P_{L}$ = 10 mW; the fiber optic laser with the collimator ($W_{0}$ = 25 μm) should be configured to $V_{s}$ = 100~400 mm/min and $P_{L}$ = 0.2~0.5 mW.

According to the screening experiment results and conclusions from the direct laser study, the parameters in Table 4 were used to manufacture four stents, matching the number of replicas in the direct laser experiment. To validate the RSM model for $S_{W}$, Equation (4) was used to calculate the theoretical value.

**Figure 5:**
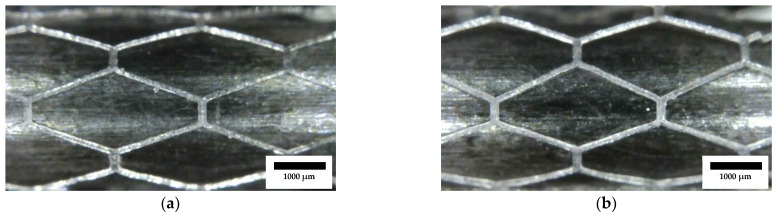
Optical microscope images from stents printed after collimator installation and decreasing the laser power: (**a**) 0.2 mW; (**b**) 0.4 mW. Scale bar is 1000 µm.

To fabricate ultrathin stents, the exposure had to be lowered. To achieve the desired $S_{W}$, the feedrate was increased to *F* = 150 mm/min (Figure 5a) for the first attempt, and the power was decreased (Figure 5b) for the second attempt. As can be seen in Figure 5, stents with a better surface finish were fabricated, eliminating the waves. In addition, with the new laser setup and machine improvements, thinner struts, and a stent thickness equal to that of commercial stents were achieved. The strut widths achieved were 70 μm, and with two layers, the stent thickness was 100 μm.

**Figure 6:**
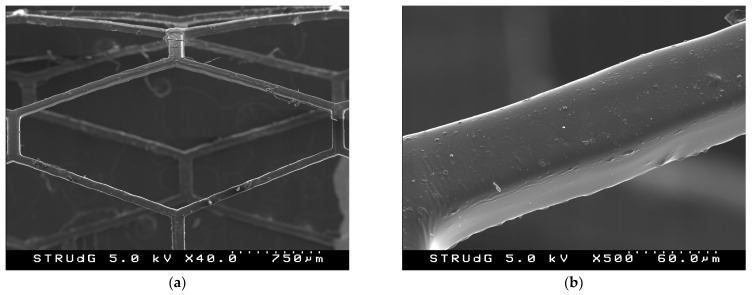

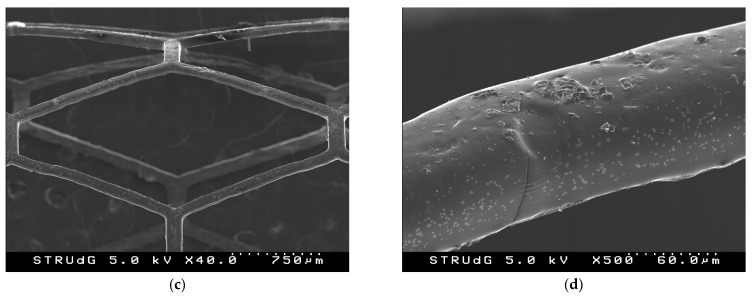
SEM images from stents printed after collimator installation: (**a**) cell detail (0.2 mW); (**b**) strut roughness, width and thickness (0.2 mW); (**c**) cell detail (0.4 mW); (**d**) strut roughness, width and thickness (0.4 mW).

SEM images (Figure 6) of the previously presented stents show a uniform and homogeneous morphology, consistent with SLA technology. The surface finish in the lowest-power case is smoother, coinciding with the lowest strut widths. Furthermore, the definition of the two dimensions of the stents is better in the 0.2 mW case, with the strut width part and the stent thickness part being very clear. In the 0.4 mW case, some bumps are observed on the surface, possibly formed by poor agitation prior to printing. Although the qualitative surface finish is close to that required to foster endothelialization [24], the stent could still be subjected to different postprocesses to improve it.

### 3.4. Differential Scanning Calorimetry

**Figure 7:**
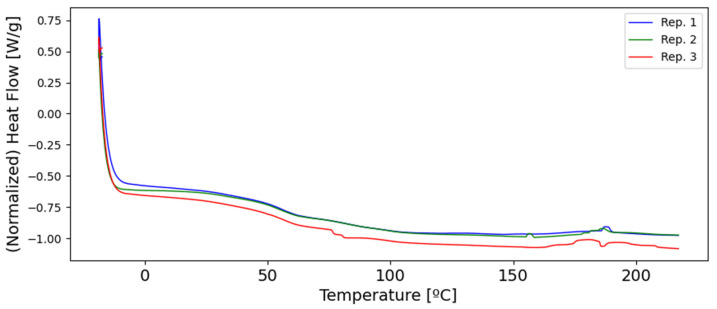
Differential Scanning Calorimetry results for 3 different stents, fabricated using the same process parameters.

To analyze the replicability and stability of the tubular SLA method, DSC testing was performed. The results from Differential Scanning Calorimetry on the whole of the analyzed samples presented a similar thermal behavior, especially between their glass transition temperature and 150 °C (Figure 7). Therefore, it can be considered a steady state of the process even with a negligible uncured residual, with a minimal value of enthalpy.

## 4. Conclusions

In this work, an innovative manufacturing method for polymeric stent (both permanent and bioresorbable) production was presented. The developed technology, based on SLA, maximally simplifies the manufacturing process; the rotary mandrel eliminates the need to use supports during printing [25] and reduces the time and amount of resin needed to produce a stent. Compared to the traditional laser cutting process, the method presented in this work allows numerous advantages, such as no surface damage, the possibility of obtaining a homogeneous molecular structure (since it is a consolidation process and not a subtraction process), better resolution, and the possibility of creating stents composed of different materials or surfaces with functionalized reliefs, among others. As demonstrated, via the ST3DT method, strut widths of less than 70 μm can be obtained, and the strut width can be controlled via the exposure (laser power and feedrate). On the other hand, a stent thickness of around 100 μm can be achieved, and the thickness can be controlled via the volume of resin and number of layers. In essence, ST3DT can fabricate a 20 mm length single-layer ultrathin stent in less than a minute using as little as 0.1 mL of resin.

Regarding the experimental results, very high printing speeds are not desirable as they agitate the resin contained in the vat, leading to decreased accuracy and the promotion of stent defect formation. The laser exposure results proved that the higher the exposure, the thicker the stent strut. The RSM model matched with the fundamental laws of stereolithography, proving that the DoE process followed is valid.

In terms of manufacturing process, further studies that analyze other trajectory strategies and different stent designs may be of interest to optimize the manufacturing process. These new strategies should involve less resin movement during the manufacturing process and should be able to increase the speed without encouraging the irregularities discussed above.

With regard to the final stent, deep studies on its mechanical behavior, surface quality, and biological response should be performed using a real biocompatible photosensible resin.

The manufacturing results obtained with this technology are promising and overcome the limitations of other previously used and available technologies. However, advances in light-curing materials available for the fabrication of class III devices are essential to characterize the mechanical properties of stents fabricated using this technology.

## 5. Patents

Patent: Reference EP3991945A1/WO2022090451A1 by Eurecat, Technology Centre of Catalonia (Cerdanyola del Vallès, Spain).

## Figures and Tables

**Table 1:** Surgical Guide components.

Name	Weight [%]
Methacrylate Monomer	25–45
Photoinitiator	1–2
Urethane Dimethacrylate	55–75

**Table 2:** Runs of the Design of Experiments.

Run	1	2	3	4	5	6	7	8	9	10	11	12	13	14	15	16
Exposure [mJ/mm^2^]	7.76	7.76	9.98	9.98	8.87	8.87	8.87	7.76	7.76	8.87	8.87	9.98	8.87	9.98	8.87	8.87
Volume [mL]	0.2	0.3	0.2	0.4	0.3	0.2	0.3	0.4	0.3	0.3	0.4	0.3	0.4	0.3	0.3	0.2
Number of layers	2	1	2	2	2	1	2	2	3	2	1	1	3	3	2	3

**Table 3:** Strut width values obtained via different methods (direct laser).

Method	Equation or Sample	Strut Width [μm]
Beer–Lambert Law	Equation (5)	199.6
Model	Equation (4)	181.8
Experimental	Average of Run 6 replicas	191.7

**Table 4:** Results after collimator installation.

$P_{L}$ [mW]	$V_{s}$ [mm/min]	*E* [mJ/mm^2^]	*V* [mL]	*L* [ut]	$S_{W}$ Model	$S_{W}$ Experimental	Error [%]
0.4	100	7.85	0.2	2	100.5 μm	92.4 μm	8.1%
0.4	100	7.85	0.2	2	100.5 μm	98.1 μm	2.4%
0.4	100	7.85	0.2	2	100.5 μm	96.3 μm	4.2%
0.4	100	7.85	0.2	2	100.5 μm	95.9 μm	4.6%

## Data Availability

The data that support the findings of this study are available from the corresponding author, A.J.G., upon reasonable request.

## Nomenclature

AM	Additive Manufacturing
ANOVA	Analysis of Variance
BBD	Box–Behnken Design
BRS	Bioresorbable Stents
DES	Drug-Eluting Stents
DIW	Direct Ink Writing
DoE	Design of Experiment
DLP	Direct Light Processing
DSC	Differential Scanning Calorimetry
IPA	Isopropyl Alcohol
FDM	Fused Deposition Modelling
PCL	Polycaprolactone
PLGA	Poly(lactic-co-glycolic acid)
PLLA	Poly(L-Lactide) Acid
SEM	Scanning Electron Microscopy
SLA	Stereolithography Assembly
ST3DT	Stereolithography 3D Tubular
RSM	Response Surface Modelling
μCLIP	micro-Continuous Liquid Interface Production
